# Efficacy, persistence and vector susceptibility to pirimiphos-methyl (Actellic® 300CS) insecticide for indoor residual spraying in Zanzibar

**DOI:** 10.1186/s13071-015-1239-x

**Published:** 2015-12-09

**Authors:** Khamis A. Haji, Narjis G. Thawer, Bakari O. Khatib, Juma H. Mcha, Abdallah Rashid, Abdullah S. Ali, Christopher Jones, Judit Bagi, Stephen M. Magesa, Mahdi M. Ramsan, Issa Garimo, George Greer, Richard Reithinger, Jeremiah M. Ngondi

**Affiliations:** Zanzibar Malaria Elimination Programme, Zanzibar, Tanzania; RTI International, Dar es Salaam, Tanzania; Liverpool School of Tropical Medicine, Liverpool, UK; President’s Malaria Initiative/United States Agency for International Development, Dar es Salaam, Tanzania; RTI International, Washington DC, USA

**Keywords:** Indoor residual spraying, Actellic 300CS, Wall surfaces, Mosquito mortality, Insecticide resistance, *Anopheles gambiae* ss, Zanzibar

## Abstract

**Background:**

Indoor residual spraying (IRS) of households with insecticide is a principal malaria vector control intervention in Zanzibar. In 2006, IRS using the pyrethroid lambda-cyhalothrine was introduced in Zanzibar. Following detection of pyrethroid resistance in 2010, an insecticide resistance management plan was proposed, and IRS using bendiocarb was started in 2011. In 2014, bendiocarb was replaced by pirimiphos methyl. This study investigated the residual efficacy of pirimiphos methyl (Actellic® 300CS) sprayed on common surfaces of human dwellings in Zanzibar.

**Methods:**

The residual activity of Actellic 300CS was determined over 9 months through bioassay tests that measured the mortality of female *Anopheles* mosquitoes, exposed to sprayed surfaces under a WHO cone. The wall surfaces included; mud wall, oil or water painted walls, lime washed wall, un-plastered cement block wall and stone blocks. Insecticide susceptibility testing was done to investigate the resistance status of local malaria vectors against Actellic 300CS using WHO protocols; Anopheline species were identified using PCR methods.

**Results:**

Baseline tests conducted one-day post-IRS revealed 100 % mortality on all sprayed surfaces. The residual efficacy of Actellic 300CS was maintained on all sprayed surfaces up to 8 months post-IRS. However, the bioassay test conducted 9 months post-IRS showed the 24 h mortality rate to be ≤80 % for lime wash, mud wall, water paint and stone block surfaces. Only oil paint surface retained the recommended residual efficacy beyond 9 months post-IRS, with mortality maintained at ≥97 %. Results of susceptibility tests showed that malaria vectors in Zanzibar were fully (100 %) susceptible to Actellic 300CS. The predominant mosquito vector species was *An. arabiensis* (76.0 %) in Pemba and *An. gambiae* (83.5 %) in Unguja.

**Conclusion:**

The microencapsulated formulation of pirimiphos methyl (Actellic 300CS) is a highly effective and appropriate insecticide for IRS use in Zanzibar as it showed a relatively prolonged residual activity compared to other products used for the same purpose. The insecticide extends the residual effect of IRS thereby making it possible to effectively protect communities with a single annual spray round reducing overall costs. The insecticide proved to be a useful alternative in insecticide resistance management plans.

## Background

The sustained implementation of key malaria control interventions by the Zanzibar Malaria Elimination Program (ZAMEP) and its partners has led to a substantial decline in malaria burden in Zanzibar, with malaria prevalence declining from >25 % in 2005 to <1 % in 2010 [1–4]. These malaria control interventions include indoor residual spraying (IRS) of households with insecticide, distribution of free long lasting insecticide treated nets (LLINs), use of rapid diagnostic tests (RDTs) and case management with artemisinin-based combination therapy (ACT) in all health facilities coupled with rigorous malaria surveillance [2, 3, 5].

IRS is a principal vector control intervention for malaria control in Zanzibar as advocated by World Health Organization (WHO) [6, 7]. Insecticide is applied on to houses’ inside walls and surfaces, which represent resting places for mosquitoes. Mosquitoes are killed upon contact with treated surfaces [8]. Through support of the U.S. President’s Malaria Initiative, IRS operations began in Zanzibar in 2006 and six blanket rounds of IRS were implemented between 2006 and 2012 in all eligible households [9].

In 2006, IRS operations used the pyrethroid lambda-cyhalothrin (ICON 10WP) [9]. In 2009, the ICON 10WP was replaced by ICON 10CS (i.e. lambda-cyhalothrin in capsule suspension rather than wettable powder) for longer residual effect on sprayed surfaces (4 to 6 months) [9]. In 2010, pyrethroid resistance against local malaria vectors was detected for the first time in Zanzibar [10]. Pyrethroid resistance is widespread and was reported by many countries in Africa [11–16]. When planning for IRS operations, WHO’s Global Plan for Insecticide Resistance Management (GPIRM) recommends rotation of insecticides with different modes of action from one year to the next [17]. Other alternative classes of insecticide recommended by WHO’s Pesticide Evaluation Scheme (WHOPES) for IRS use include bendiocarb, (a carbamate) and pirimiphos methyl (p-methyl; an organophosphate) that are both acetylcholinesterase inhibitors and could be used in rotation for insecticide resistance management [18, 19]. Following the detection of pyrethroid resistance in Zanzibar, an insecticide resistance management plan was developed and IRS with bendiocarb started in 2012 in line with the GPIRM-recommended rotation approach [9, 10]. Bendiocarb was planned to be used for three IRS rounds to the end of 2013 [9]. However, bendiocarb has a short residual life on sprayed surfaces (i.e. 2 to 6 months), which leads to increased IRS operational costs due to the requirement of multiple IRS rounds [20]. Hence, in 2014, p-methyl (Actellic® 300CS, Syngenta Crop protection, Switzerland) was selected for use in Zanzibar, as the insecticide has a longer residual life than other insecticides [21].

A number of studies have shown Actellic 300CS to have good residual efficacy for use in IRS operations [21]. The efficacy and duration of activity of p-methyl 300 CS was compared to other insecticides used for IRS against malaria vectors in small-scale and large-scale field trials conducted in Benin, Ethiopia, Gambia, India, Senegal, South Africa, Tanzania, Viet Nam, Ghana and Zambia [21–25]. Varying residual efficacy of p-methyl 300 CS has been reported ranging between 3 and 9 months on various surfaces [21].

Monitoring of insecticides is essential to determine the periods that it remains effective in interrupting malaria transmission and schedule when to re-spray. The effectiveness of an IRS operation is influenced by the residual efficacy of the insecticide used to spray the wall surfaces [7]. According to WHOPES criteria, an insecticide is considered to have adequate residual efficacy if -over a prolonged period of time- it elicits ≥80 % mosquito mortality, 24 h post exposure on sprayed surfaces [19]. Increased or sustained residual efficacy ensures that the population at risk of malaria is protected from malaria infection during peak transmission; residual efficacy can also affect IRS operational costs if multiple IRS rounds are not required.

There are no documented studies on the influence of different wall substrate on the residual efficacy of Actellic 300CS for IRS use in Zanzibar. This study investigated residual efficacy of p-methyl sprayed on common surfaces of human dwellings in Zanzibar as well as susceptibility status of local malaria vectors against this insecticide.

## Methods

### Study site

**Fig. 1:**
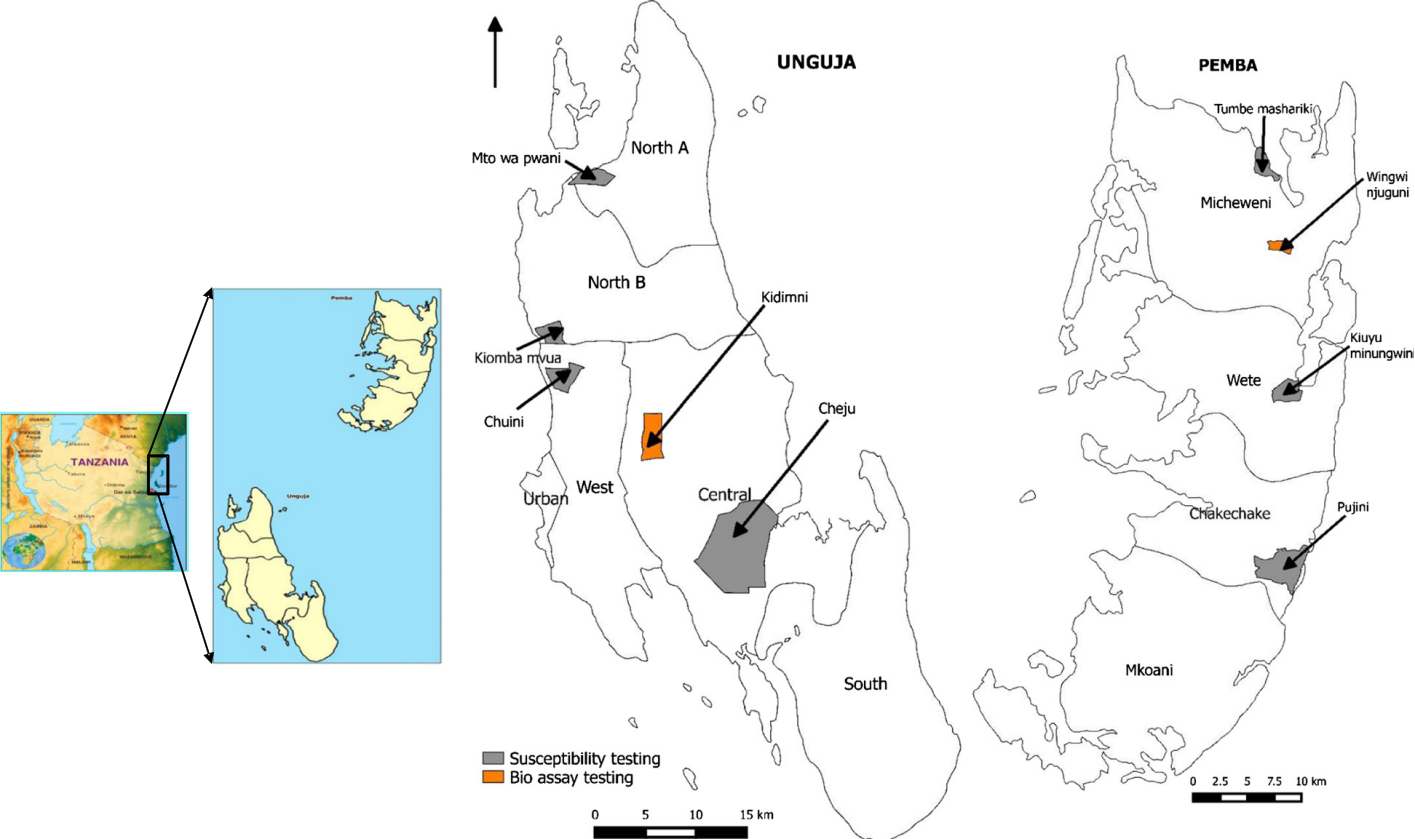
Map of Zanzibar showing the study sites for bioassay and susceptibility testing

Monthly bioassays on the efficacy of Actellic 300CS treated surfaces was undertaken in two sites in Zanzibar: Kidimni in Unguja and Njuguni in Pemba (Fig. 1). A susceptible, laboratory reared *An gambiae* R-70 strain was used for the exposures. Local malaria vector susceptibility status to Actellic 300CS was determined using F1 *Anopheles gambiae* s.l that had emerged from wild larvae collected from various sites in Unguja (Chuini, Cheju, Kiombamvua and Mto wa Pwani) and Pemba (Tumbe, Minungwini and Pujini). Bioassays were conducted at ZAMEP laboratories in Unguja and Mkoroshoni Insectary in Pemba, from June 2013 –February 2014.

### Study design

The bio-efficacy testing was a longitudinal study aimed at collecting information from selected study sites on monthly basis for a 9-month period. The susceptibility testing was designed to be a cross-sectional study that collected information at a single point in time. All testing was conducted as per WHO test procedures for insecticide resistance monitoring [26].

### Spraying of surfaces with Actellic 300CS

Actellic 300CS was sprayed on all test wall surfaces at the recommended dose of one gram of active ingredient/m^2^ using a Hudson® X-pert spray pump [20]. The different surfaces that were sprayed included, oil and water painted walls, un-plastered cement block wall, mud plastered wall, lime washed wall and stone blocked wall. The spraying was done by an experienced spray operator using a Hudson® X-pert compression sprayer (Hudson Manufacturing Company) with a flat nozzle as recommended for IRS by WHO [27].

Since all the surfaces sprayed were flat, a nozzle, which produces a flat swathe, was used throughout the spraying operation. One bottle containing 833 ml of Actellic 300CS was mixed in 10 l of portable water into the Hudson sprayer that was pressurized to the optimal range of 35–55 (psi) as per manufacturers’ instructions. A distance of 45 cm from the nozzle tip to the surface being sprayed was maintained during spraying. At this distance, the width of the swathe at the point of impact was 75 cm. A 5 cm overlap was maintained between the swathes to make sure that no wall surface was left without insecticide. Preparations undertaken in households prior to the spraying included: removal of movable household contents; covering of non-movable contents with plastic sheets; and removal of wall coverings and curtains. Household occupants were instructed to stay outdoors during and for at least 2 h post spraying.

### Mosquito rearing

An insecticide susceptible strain of *Anopheles gambiae* s.s (R-70) for bio-efficacy testing was reared in the ZAMEP insectaries in Unguja and Mkoroshoni in Pemba. Mosquitoes were maintained under controlled conditions in the insectary [28]. Wild collected *Anopheles gambiae* s.l*.* larva from different locations of Unguja (Chuini, Cheju and Kiombamvua) and Pemba (Tumbe, Minungwini and Pujini) were reared to adults and tested for their susceptibility to Actellic 300CS. The rearing and testing was conducted at ZAMEP insectary in Unguja and Mkoroshoni in Pemba as per the WHO guidelines [19].

### Residual efficacy testing

Two to five day-old unfed females of *An. gambiae* s.s. (R-70 strain) from ZAMEP insectary were taken out of a cage using mouth-operated aspirators. The mosquitoes were gently blown into each bioassay cone fixed on the treated wall surfaces using masking tape. Cotton wool was used to plug the cone opening to stop mosquitoes from escaping. Each test batch introduced into each cone was made of ten female mosquitoes that were exposed for 30 min. At the end of exposure time, the mosquitoes were aspirated out of the cone and gently blown into a holding paper cup for a 24 h holding period. Knock-down (KD_30_) was scored immediately at the end of exposure time of 30 min and mortality was scored 24 h post exposure. Mosquitoes were classified as dead if they were immobile or unable to stand or fly in a coordinated way. Exposure on each treated surface was replicated thrice for each monthly time point post-IRS; an untreated surface of each type was used as control.

### Susceptibility testing

Twenty five unfed females *Anopheles gambiae* s.l (age range between 2 and 5 days) were exposed to 0.25 % Actellic 300CS impregnated paper lined into a WHO susceptibility tube for one hour and then removed. Exposed mosquitoes were transferred into a clean holding tube lined with a blank paper and provided with 10 % sugar solution. Mortality was scored at the end of a 24 h holding period [28]. Members of the *An. gambiae* s.l. species complex were identified using allele-specific PCR [29].

### Data analysis

Data were analyzed using Microsoft Excel® (Microsoft Corporation, Redmond, WA). The results were presented using line graphs that show the rate of insecticide decay versus number of days post-IRS. Mosquito mortality was used as an indicator of decreasing insecticide residual efficacy on a given tested substrate.

### Ethical consideration

Consent was sought from head of households for all house structures sprayed for bio-efficacy testing. This study was undertaken as part of Zanzibar-wide IRS operations; therefore, it was not eligible for ethical clearance. Permission to publish these data was granted by the Zanzibar Medical Research Ethical Committee (ZAMREC).

## Results

### Residual efficacy of Actellic 300CS

**Fig. 2:**
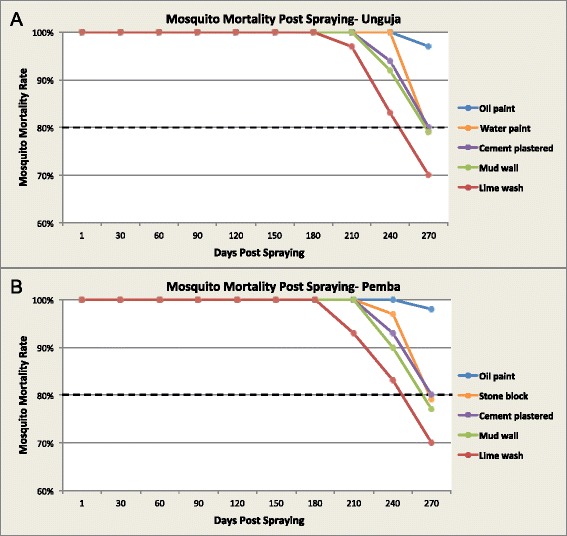
Mosquito mortality rate on wall substrates (**a**) in Unguja and (**b**) in Pemba throughout the study period. The dashed line represents the WHO threshold of 80 % mosquito mortality rate

The residual efficacy of Actellic 300CS on tested substrates was monitored for a period of 9 months. Different linear patterns were observed among the substrates tested in both Unguja and Pemba as shown in Fig. 2a and b respectively and Table 1. Baseline bioassay tests conducted one-day post-IRS revealed 100 % mortality on all sprayed surfaces. The high residual efficacy of Actellic 300CS was maintained on all sprayed surfaces up to 8 months post-IRS. However, the bioefficacy tests conducted at 9 months post-IRS showed 24 h mortality to be ≤80 % for lime wash, mud wall, water paint and stone block surfaces. Only oil paint surfaces retained the recommended residual efficacy beyond 9 months post-IRS, with mortality being 97 and 98 % for mosquitoes exposed to sprayed surfaces in Unguja and Pemba, respectively.

**Table 1:** Mortality rate (%) of *Anopheles gambiae* s.s. exposed to different surfaces sprayed with a micro-encapsulated formulation of pirimiphos-methyl (Actellic 300CS) in Unguja and Pemba

Island	Substrate	Days post-IRS									
		1	30	60	90	120	150	180	210	240	270
Unguja (Kidimni)	Water paint	100 %	100 %	100 %	100 %	100 %	100 %	100 %	100 %	100 %	79.0 %
	Cement plastered	100 %	100 %	100 %	100 %	100 %	100 %	100 %	100 %	94.0 %	80.0 %
	Oil paint	100 %	100 %	100 %	100 %	100 %	100 %	100 %	100 %	100 %	97.0 %
	Lime wash	100 %	100 %	100 %	100 %	100 %	100 %	100 %	97.0 %	83.0 %	70.0 %
	Mud wall	100 %	100 %	100 %	100 %	100 %	100 %	100 %	100 %	92.0 %	79.0 %
	Control	0 %	0 %	0 %	0 %	0 %	0 %	0 %	0 %	0 %	0 %
Pemba (Ungujuni)	Stone block	100 %	100 %	100 %	100 %	100 %	100 %	100 %	100 %	97.0 %	79.0 %
	Cement plastered	100 %	100 %	100 %	100 %	100 %	100 %	100 %	100 %	93.0 %	80.0 %
	Oil paint	100 %	100 %	100 %	100 %	100 %	100 %	100 %	100 %	100 %	98.0 %
	Lime wash	100 %	100 %	100 %	100 %	100 %	100 %	100 %	93.0 %	83.0 %	70.0 %
	Mud wall	100 %	100 %	100 %	100 %	100 %	100 %	100 %	100 %	90.0 %	77.0 %
	Control	0 %	0 %	0 %	0 %	0 %	0 %	0 %	0 %	0 %	0 %

### Vector susceptibility

Table 2 summarizes the findings of the vector susceptibility testing that was undertaken on local malaria vectors (*Anopheles gambiae* s.l) against Actellic 300CS in both Unguja and Pemba islands. All *Anopheles* mosquitoes tested were 100 % susceptible to Actellic 300CS.

**Table 2:** Susceptibility of *Anopheles gambiae* s.l. to the micro-encapsulated formulation of pirimiphos-methyl (Actellic 300CS) in Unguja and Pemba

Island	Site	No. of mosquitoes tested	Mortality after 24 h (%)
Pemba	Tumbe	100	100 %
	Minungwini	200	100 %
	Pujini	100	100 %
Unguja	Chuini	100	100 %
	Cheju	100	100 %
	Kiombamvua	100	100 %
	Mtowapwani	100	100 %

### *Anopheles gambiae* s.l. sibling species

For susceptibility testing, 400 wild-caught *Anopheles gambiae* s.l. mosquitoes were used. The vector species composition of these mosquitos is shown in Table 3. The predominant mosquito vector species was *An. arabiensis* (76.0 %) in Pemba and *An. gambiae* s.s (83.5 %) in Unguja; *An. merus* was more common in Pemba (20.0 %) compared to Unguja (2.5 %).

**Table 3:** Distribution of vector species by island

Island	Site	Number of mosquitoes tested	Species composition		
			*An. gambiae*	*An. arabiensis*	*An. merus*
Pemba	Tumbe	50	16.0 %	6.0 %	78.0 %
	Pujini	50		100.0 %	
	Minungwini	100		99.0 %	1.0 %
	Sub-total	200	4.0 %	76.0 %	20.0 %
Unguja	Cheju	50	66.0 %	24.0 %	10.0 %
	Chuini	50	72.0 %	28.0 %	
	Kiomba Mvua	100	98.0 %	2.0 %	
	Sub-total	200	83.5 %	14.0 %	2.5 %
Total		400	43.8 %	45.0 %	11.3 %

## Discussion

This study investigated the duration of residual efficacy of Actellic 300CS sprayed on common surfaces of human dwellings in Zanzibar and vector susceptibility to Actellic 300CS among local malaria vectors. The results of the bioassay testing showed that Actellic 300CS remains effective for up to eight months post-IRS on a range of different wall surfaces. Susceptibility testing showed that local malaria vectors in Zanzibar are 100 % susceptible to this insecticide. The predominant vector species were *An. arabiensis* in Pemba and *An. gambiae* s.s in Unguja.

The current findings on the duration of residual efficacy are comparable to previous studies conducted in Benin [25], Tanzania [23], central Côte d’Ivoire [30], Zambia [24], Ethiopia and Senegal [21]. These studies showed that the duration of effective residual efficacy of Actellic 300CS ranged between 2 and 11 months on various surfaces by inflicting ≥80 % mortality of *An. gambiae*. The microencapsulated form of p-methyl demonstrates longer residual activity against malaria vectors than the 500 EC formulation that has been shown to have a duration of effective residual activity of 2–3 months [31, 32].

Recent studies in Zanzibar have documented widespread malaria vector resistance to pyrethroids; however, malaria vectors are still susceptible to carbamates (bendiocarb) [10, 33]. Vector susceptibility tests showed that Actellic 300 CS was highly effective against the local malaria vectors, inflicting 100 % mortality, 24 h post-exposure. These findings suggest that insecticide rotation with microencapsulated p-methyl could be beneficial in managing insecticide resistance and should be recommended for use in IRS operations in Zanzibar. Tested insecticide would serve as an alternative and provide prolonged control of pyrethroid-resistant *An. gambiae* with only a single IRS round per year. It is also possible that use of Actellic 300 CS could lead to reversal of pyrethroid resistance by eliminating resistant vector populations; thus, allowing for potential re-introduction of pyrethroids for use in IRS operations in the future [10, 33].

The predominant vector species were *An. arabiensis* in Pemba and *An. gambiae* s.s in Unguja. These findings are in line with previous studies that demonstrate that *An. arabiensis* is the main malaria vector on Pemba Island [10, 33]. This also has implications on vector control interventions [34]. The *An. arabiensis* is known to feed on both indoors and outdoors, on humans and non-human hosts and can rest outdoors unlike the *An. gambiae s.s* that is more endophilic and anthropophilic [10]. Thus, in addition to indoor interventions such as LLINs and IRS, more tools are required for preventing outdoor-biting leading to residual transmission, especially in Pemba.

## Conclusion

Based on our findings Actellic 300CS is highly effective and appropriate for IRS in Zanzibar as it showed prolonged residual activity. Susceptibility testing showed that local malaria vectors in Zanzibar are 100 % susceptible to the insecticide. Thus, the encapsulated formulation of pirimiphos-methyl represents a useful alternative to other insecticides for resistance management in Zanzibar.

## Abbreviations

ZAMEP: Zanzibar Malaria Elimination Program
IRS: Indoor residual spraying
LLINS: Long lasting insecticide treated nets
ACT: Artemisinin-based combination therapy
RDTs: Rapid diagnostic tests
WHO: World Health Organization
WHOPES: World Health Organization Pesticide Evaluation Scheme
p-methyl: Pirimiphos methyl
ZAMREC: Zanzibar Medical Research Ethical Committee
